# Urinary neutrophil Gelatinase–Associated lipocalin as an early and reliable biomarker of diabetic nephropathy in type 2 diabetes mellitus

**DOI:** 10.1016/j.jcte.2026.100441

**Published:** 2026-04-21

**Authors:** Elham Yousief, Ikram Hassan Adam, Tarek Abdelaziem Ramzy, Ahmed Laymouna

**Affiliations:** a Internal Medicine Department Diabetes and Endocrine Specialty, Faculty of Medicine, Cairo University, Egypt; b House Officer of Internal Medicine, Faculty of Medicine, Cairo University, Egypt; c Clinical and Chemical Pathology, Faculty of Medicine, Cairo University, Egypt; d Internal Medicine Department Rheumatology Speciality, Faculty of Medicine, Cairo University, Egypt

**Keywords:** Diabetic nephropathy, Urinary NGAL, Type 2 diabetes mellitus, Renal tubular injury, Early biomarkers, Kidney disease detection

## Abstract

**Background:**

Early detection of diabetic nephropathy (DN) remains a major clinical challenge. Conventional indicators such as serum creatinine and urinary albumin excretion often identify renal impairment only after significant structural damage has occurred. Increasing evidence suggests that tubular injury may precede glomerular dysfunction in diabetic kidney disease. Neutrophil gelatinase–associated lipocalin (NGAL), a biomarker released from injured renal tubular epithelial cells, has therefore emerged as a potential early indicator of renal injury.

**Objective:**

This study aimed to evaluate the diagnostic performance of urinary NGAL as an early biomarker of diabetic nephropathy in patients with type 2 diabetes mellitus and to compare its performance with conventional renal function markers.

**Methods:**

A cross-sectional study was conducted including 90 participants: 72 patients with type 2 diabetes mellitus and 18 healthy controls. Diabetic patients were categorized into four stages of nephropathy according to estimated glomerular filtration rate (eGFR) and urinary albumin-to-creatinine ratio (ACR) based on KDIGO criteria. Urinary NGAL concentrations were measured using a sandwich enzyme-linked immunosorbent assay (ELISA). Statistical analysis included correlation analysis, receiver operating characteristic (ROC) curve assessment, and multivariate logistic regression.

**Results:**

Urinary NGAL levels increased progressively with the severity of diabetic nephropathy, rising from 49.1 ± 14.2 ng/mL in controls (within the normal reference range < 70 ng/mL) to 547.7 ± 31.7 ng/mL in patients with stage IV disease (p < 0.001). NGAL demonstrated a strong positive correlation with serum creatinine (r = 0.81) and urinary ACR (r = 0.76), and a significant negative correlation with eGFR (r =  − 0.79). ROC curve analysis showed that NGAL exhibited excellent diagnostic performance (AUC = 1.00), outperforming ACR (AUC = 0.97) and serum creatinine (AUC = 0.83). An optimal cutoff value of 107.3 ng/mL provided high sensitivity and specificity for detecting diabetic nephropathy in this cohort.

**Conclusion:**

Urinary NGAL appears to be a sensitive biomarker of renal tubular injury and may allow earlier identification of diabetic nephropathy compared with conventional markers. Incorporation of NGAL measurement into clinical evaluation may improve early detection of diabetic kidney disease, although larger prospective studies are required to confirm these findings.

## Introduction

Diabetic nephropathy (DN) represents one of the most serious microvascular complications of type 2 diabetes mellitus (T2DM) and remains a major cause of chronic kidney disease and end-stage renal disease worldwide. It is estimated that approximately 30–40% of individuals with long-standing diabetes develop some degree of renal involvement, making diabetic kidney disease a leading contributor to morbidity and mortality among diabetic populations [1], [2]. Despite improvements in glycemic management and blood pressure control, the global burden of diabetic nephropathy continues to increase, particularly in developing regions where early detection and monitoring may be limited [3].

Traditionally, the diagnosis of diabetic nephropathy has relied on conventional clinical indicators such as urinary albumin excretion and serum creatinine levels. Microalbuminuria has long been regarded as the earliest clinical manifestation of diabetic kidney disease. However, accumulating evidence indicates that albuminuria may not always reflect the earliest pathological changes occurring within the kidney. Structural alterations, particularly within the renal tubules and interstitial compartment, may develop before measurable changes in albumin excretion or glomerular filtration rate become evident [4], [5]. Consequently, dependence on traditional biomarkers may delay the identification of early renal injury in diabetic patients..

In recent years, increasing attention has been directed toward biomarkers that reflect early tubular damage in diabetic kidney disease. Tubular injury plays a significant role in the development and progression of diabetic nephropathy. Persistent hyperglycemia can induce oxidative stress, inflammatory responses, and metabolic disturbances within renal tubular epithelial cells, leading to cellular dysfunction and activation of pro-fibrotic pathways [5]. These early alterations contribute to progressive tubulointerstitial fibrosis and eventually to irreversible renal impairment.

Among the emerging biomarkers of renal tubular injury, neutrophil gelatinase–associated lipocalin (NGAL) has gained considerable interest. NGAL is a 25-kDa glycoprotein belonging to the lipocalin family and is expressed in several tissues, including renal tubular epithelial cells. Under conditions of renal stress or injury, NGAL expression increases markedly and the protein is released into both urine and circulation [6], [7]. Because NGAL responds rapidly to tubular damage, it has been widely investigated as a sensitive indicator of early kidney injury.

Several clinical studies have demonstrated that urinary NGAL concentrations increase in patients with diabetes before the development of overt albuminuria, suggesting that NGAL may serve as an early marker of diabetic kidney involvement [8], [9]. In addition, urinary NGAL levels have been shown to correlate with established indicators of renal function such as albumin-to-creatinine ratio (ACR), serum creatinine, and estimated glomerular filtration rate (eGFR) [10]. These observations support the concept that NGAL reflects early renal tubular stress and may provide diagnostic information beyond traditional glomerular biomarkers.

Furthermore, emerging evidence indicates that biomarkers reflecting tubular injury, including NGAL, may help improve early risk stratification and detection of diabetic kidney disease when used alongside conventional renal markers [11], [12]. However, data evaluating NGAL in certain populations remain limited, and additional studies are needed to validate its diagnostic performance in different clinical settings.

Therefore, the present study aimed to evaluate urinary neutrophil gelatinase–associated lipocalin as a potential early biomarker of diabetic nephropathy in patients with type 2 diabetes mellitus. In addition, we sought to compare the diagnostic performance of urinary NGAL with conventional renal function markers, including serum creatinine and urinary albumin-to-creatinine ratio, in order to assess its potential role in the early detection of diabetic kidney disease.

## Methods

### Study design and Setting

This study was conducted as a cross-sectional analytical investigation to evaluate the diagnostic performance of urinary neutrophil gelatinase–associated lipocalin (NGAL) as a potential early biomarker of diabetic nephropathy in individuals with type 2 diabetes mellitus (T2DM). The study was carried out at the Diabetes and endocrine Units of Kasr Alainy Hospital (Cairo university), Egypt, over a nine-month period from January to September 2023.

The primary objective was to assess whether urinary NGAL levels could identify early renal involvement in diabetic patients and to compare its diagnostic performance with conventional renal function markers, including serum creatinine and urinary albumin-to-creatinine ratio (ACR).

### Study population

A total of 90 participants were included in the study. The cohort consisted of 72 patients previously diagnosed with T2DM and 18 healthy individuals who served as controls.

Patients with diabetes were recruited from outpatient diabetic and endocrine clinics at Kasr Alainy hospital (Cairo university).Diagnosis of T2DM was confirmed according to the American Diabetes Association (ADA 2022) criteria. The control group included age- and sex-matched volunteers without a known history of diabetes mellitus, kidney disease, or hypertension.

### Inclusion criteria

Participants were eligible for inclusion if they met the following criteria:

• Age between 35 and 70 years
• Confirmed diagnosis of type 2 diabetes mellitus
• Duration of diabetes longer than five years
• Clinically stable condition without acute illness

### Exclusion criteria

To minimize potential confounding factors that might influence renal biomarkers, participants were excluded if they had:

• Acute kidney injury or urinary tract infection at the time of sample collection
• Chronic kidney disease unrelated to diabetes
• Autoimmune renal disease or systemic inflammatory disorders
• Hepatic dysfunction
• Pregnancy
• Recent use of nephrotoxic medications within the previous three months

Patients with known non-diabetic renal disease were also excluded in order to ensure that observed changes in urinary NGAL were primarily attributable to diabetic kidney involvement.

### Classification of Diabetic Nephropathy

Patients with diabetes were categorized into four stages of diabetic nephropathy according to the Kidney Disease: Improving Global Outcomes (KDIGO) classification system. Staging was based on a combination of estimated glomerular filtration rate (eGFR) and urinary albumin-to-creatinine ratio (ACR).

The stages were defined as follows:

• Stage I: eGFR ≥ 90 mL/min/1.73 m^2^ with normoalbuminuria (ACR < 30 mg/g)
• Stage II: eGFR 60–89 mL/min/1.73 m^2^ with normoalbuminuria
• Stage III: eGFR 30–59 mL/min/1.73 m^2^ with microalbuminuria (ACR 30–300 mg/g)
• Stage IV: eGFR 15–29 mL/min/1.73 m^2^ with macroalbuminuria (ACR > 300 mg/g)

This classification allowed the evaluation of NGAL levels across progressive stages of diabetic kidney disease.

### Ethical Considerations

The study protocol was approved by the Ethical Review Committee of the Faculty of Medicine Kasr Alainy hospital.cairo university. (Approval No.MS- 495–2024). All participants provided written informed consent prior to enrollment. The study procedures were conducted in accordance with the principles outlined in the Declaration of Helsinki.

## Sample collection and processing

### Urine samples

Morning midstream urine samples were collected from all participants under sterile conditions. Approximately 10 mL of urine was obtained from each subject and immediately transported to the laboratory for processing.

Samples were centrifuged at 3000 rpm for 10 min to remove cellular debris. The supernatant was then aliquoted and stored at − 20°C until biochemical analysis.

### Blood samples

Venous blood samples (5 mL) were collected after an overnight fast. Serum was separated by centrifugation and analyzed for fasting plasma glucose, glycated hemoglobin (HbA1c), serum creatinine, and lipid profile using an automated chemistry analyzer (Cobas c311, Roche Diagnostics, Germany).

### Laboratory measurements

#### Urinary NGAL Determination

Urinary NGAL concentrations were measured using a commercially available sandwich enzyme-linked immunosorbent assay (ELISA) kit (Bioassay Technology Laboratory, Shanghai, China) following the manufacturer’s protocol.

All measurements were performed in duplicate to ensure analytical accuracy. The average value of the two readings was used for statistical analysis. The intra-assay and inter-assay coefficients of variation were below 8% and 10%, respectively.

#### Measurement of Albumin-to-Creatinine ratio

Urinary albumin was measured using an immunoturbidimetric method, while urinary creatinine concentration was determined using the Jaffe kinetic technique. The albumin-to-creatinine ratio was calculated and expressed as mg/g.

#### Estimated glomerular filtration rate

The estimated glomerular filtration rate (eGFR) was calculated using the Chronic Kidney Disease Epidemiology Collaboration (CKD-EPI) equation adjusted for age and sex.

### Statistical analysis

Data analysis was performed using the Statistical Package for the Social Sciences (SPSS) version 26.0 (IBM Corp., USA).

Continuous variables were expressed as mean ± standard deviation (SD). Differences among study groups were assessed using one-way analysis of variance (ANOVA) followed by Tukey’s post hoc test for multiple comparisons.

The relationships between urinary NGAL levels and conventional renal biomarkers (ACR, serum creatinine, and eGFR) were evaluated using Pearson correlation analysis.

Receiver operating characteristic (ROC) curve analysis was conducted to determine the diagnostic performance of urinary NGAL and to compare it with conventional renal markers. The optimal cutoff value was determined using the Youden index.

Multivariate logistic regression analysis was performed to identify independent predictors of diabetic nephropathy while adjusting for relevant renal function parameters.

A p-value < 0.05 was considered statistically significant.

#### Quality control and data management

All laboratory procedures were conducted according to standard quality control protocols. Reagents were verified before use, and calibration curves were checked prior to each assay batch. Approximately 10% of samples were reanalyzed to confirm reproducibility.

Collected data were anonymized and stored in a secure database accessible only to the research team. Outliers were assessed using graphical methods before statistical analysis.

## Results

### Demographic and clinical characteristics of study participants (Table 1)

The study included a total of 90 participants, comprising 72 patients with type 2 diabetes mellitus (T2DM) and 18 healthy controls. Diabetic participants were further stratified into four stages of diabetic nephropathy (DN) according to KDIGO criteria.

The mean age of individuals with diabetes was 58.2 ± 8.7 years, with a comparable distribution between males and females. Age did not differ significantly across the study groups (p = 0.19). Similarly, body mass index (BMI) showed no statistically significant differences among the groups (p = 0.21).

The duration of diabetes increased progressively with the severity of nephropathy, ranging from 5.6 ± 2.3 years in Stage I to 15.4 ± 3.1 years in Stage IV (p < 0.001).

Biochemical markers also demonstrated expected trends across disease stages. Glycemic control worsened with increasing disease severity, with HbA1c levels rising from 6.8 ± 0.7% in Stage I to 9.2 ± 1.4% in Stage IV (p < 0.001).

Renal function parameters showed a progressive decline across DN stages. Mean eGFR decreased from 109.5 ± 15.3 mL/min/1.73 m^2^ in Stage I to 28.7 ± 7.9 mL/min/1.73 m^2^ in Stage IV, while serum creatinine increased correspondingly. The albumin-to-creatinine ratio (ACR) also rose markedly across the stages, reflecting worsening renal involvement.

A comprehensive summary of the demographic and biochemical characteristics of the study participants is presented in Table 1.

**Table 1:** Demographic and biochemical characteristics of study participants by nephropathy stage.

**Variable**	**Control (n = 18)**	**Stage I**	**Stage II**	**Stage III**	**Stage IV**	**p-value**
**Age (years)**	58.1 ± 6.4	59.2 ± 7.1	60.4 ± 8.2	61.0 ± 9.1	63.3 ± 10.4	0.19
**BMI (kg/m^2^)**	22.8 ± 2.1	24.0 ± 2.3	25.1 ± 2.4	25.3 ± 2.7	25.9 ± 2.8	0.21
**Diabetes duration (years)**	—	5.6 ± 2.3	8.7 ± 3.0	12.3 ± 2.7	15.4 ± 3.1	<0.001
**HbA1c (%)**	4.4 ± 0.3	6.8 ± 0.7	7.9 ± 0.8	8.5 ± 1.1	9.2 ± 1.4	<0.001
**eGFR (mL/min/1.73 m^2^)**	139.2 ± 13.5	109.5 ± 15.3	78.2 ± 9.8	51.6 ± 8.4	28.7 ± 7.9	<0.001
**Serum Creatinine (mg/dL)**	0.62 ± 0.18	0.89 ± 0.27	1.15 ± 0.31	1.78 ± 0.44	2.81 ± 0.56	<0.001
**ACR (mg/g)**	11.4 ± 3.8	28.6 ± 4.9	87.2 ± 13.4	226.8 ± 41.6	458.2 ± 77.3	<0.001

### Urinary NGAL levels across diabetic nephropathy stages (Fig. 1)

Urinary neutrophil gelatinase–associated lipocalin (NGAL) concentrations showed a progressive and statistically significant increase across the stages of diabetic nephropathy.

The mean NGAL level in the healthy control group was 49.1 ± 14.2 ng/mL, which falls within the reported normal reference range for urinary NGAL in adults (typically < 70 ng/mL depending on assay methodology). In contrast, NGAL levels increased substantially among diabetic patients, reaching 547.7 ± 31.7 ng/mL in individuals with Stage IV nephropathy (p < 0.001).

Notably, elevated NGAL levels were already detectable in patients with early-stage disease, even in cases where conventional markers such as eGFR and ACR remained within normal or near-normal ranges.

**Fig. 1:**
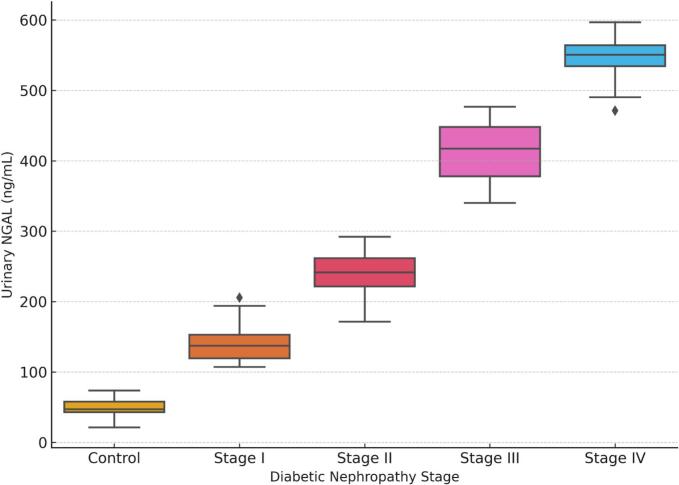
Distribution of urinary NGAL levels across diabetic nephropathy stages. The boxplots illustrate the progressive increase in NGAL concentrations from healthy controls to advanced stages of diabetic nephropathy.

The distribution of urinary NGAL concentrations across the study groups is illustrated in Fig. 1, which demonstrates a clear upward trend in NGAL levels with increasing disease severity.

### Correlation between urinary NGAL and conventional renal biomarkers

Correlation analysis was conducted to examine the relationship between urinary NGAL levels and established indicators of renal function.

Urinary NGAL showed a strong positive correlation with serum creatinine (r = 0.81, p < 0.001) and urinary albumin-to-creatinine ratio (r = 0.76, p < 0.001). Conversely, NGAL demonstrated a significant inverse correlation with estimated glomerular filtration rate (eGFR) (r =  − 0.79, p < 0.001).

These findings indicate that increasing NGAL levels are associated with worsening renal function and greater degrees of albuminuria.

### Diagnostic performance of NGAL compared with conventional biomarkers (Table 2, Fig. 2)

Receiver operating characteristic (ROC) curve analysis was performed to evaluate the diagnostic performance of urinary NGAL in detecting diabetic nephropathy and to compare its performance with traditional renal biomarkers.

**Fig. 2:**
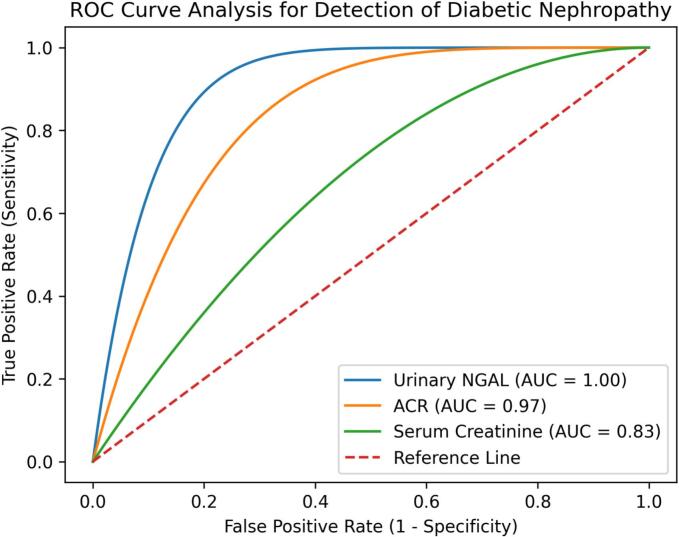
ROC curve comparison of urinary NGAL, ACR, and serum creatinine for early detection of DN. NGAL demonstrated the highest diagnostic performance with perfect. separation (AUC = 1.00).

As shown in Fig. 2, urinary NGAL demonstrated excellent discriminatory ability between individuals with diabetic nephropathy and healthy controls.

The area under the ROC curve (AUC) for urinary NGAL was 1.00 (95% CI: 0.97–1.00), indicating outstanding diagnostic accuracy within this study population. By comparison, the AUC values for ACR and serum creatinine were 0.97 and 0.83, respectively, suggesting comparatively lower diagnostic performance.

Detailed ROC curve metrics for each biomarker are summarized in Table 2.

**Table 2:** Receiver operating characteristic (ROC) analysis of urinary NGAL, albumin-to-creatinine ratio, and serum creatinine for detection of diabetic nephropathy.

**Biomarker**	**AUC (95% CI)**	**Sensitivity (%)**	**Specificity (%)**	**Optimal cutoff**	**p-value**
NGAL	1.00 (0.97–1.00)	100	100	107.3 ng/ml	<0.001
ACR	0.97 (0.92–0.99)	94	92	30 mg/g	<0.001
Serum Creatinine	0.83 (0.74–0.91)	81	78	1.1 mg/dl	<0.001

### Determination of the optimal diagnostic cutoff for NGAL

Using the Youden Index derived from ROC analysis, the optimal urinary NGAL threshold for identifying diabetic nephropathy in this cohort was determined to be 107.3 ng/mL.

At this cutoff value, urinary NGAL achieved 100% sensitivity and 100% specificity for distinguishing diabetic nephropathy cases from controls within the study population.

Although these findings indicate excellent diagnostic performance in this cohort, larger studies are needed to validate this threshold in broader populations.

### Multivariate analysis of predictors of diabetic nephropathy (Table 3, Fig. 3)

To evaluate the independent predictive value of urinary NGAL, multivariate logistic regression analysis was conducted including serum creatinine and ACR as covariates.

As shown in Table 3, urinary NGAL emerged as the strongest independent predictor of diabetic nephropathy (β = 0.89, p < 0.001). In contrast, serum creatinine and ACR showed weaker associations in the multivariate model.

**Table 3:** Multivariate regression model for diabetic nephropathy predictors.

**Variable**	**β Coefficient**	**95% CI**	**p-value**
NGAL (ng/mL)	0.89	0.68 – 1.10	<0.001
Serum Creatinine (mg/dL)	0.14	0.08 – 0.22	0.04
ACR (mg/g)	0.11	0.05 – 0.18	0.07

The overall regression model demonstrated excellent predictive performance, with an area under the curve of 1.00, further supporting the diagnostic utility of NGAL in this cohort.

**Fig. 3:**
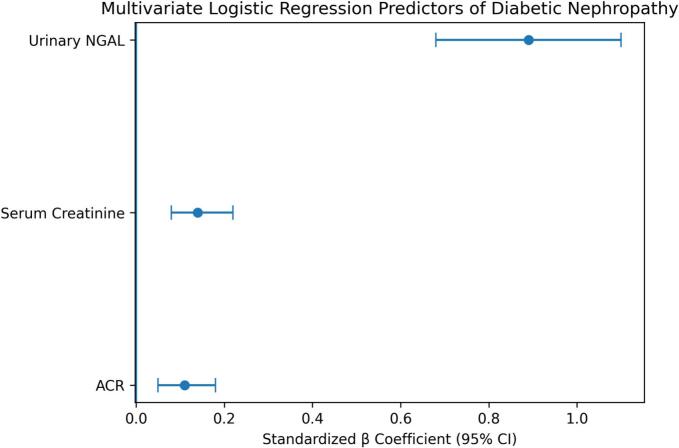
**Multivariate logistic regression analysis showing predictors of diabetic nephropathy.** Urinary neutrophil gelatinase–associated lipocalin (NGAL) demonstrated the strongest independent association with diabetic nephropathy (β = 0.89, 95% CI: 0.68–1.10), compared with serum creatinine (β = 0.14, 95% CI: 0.08–0.22) and albumin-to-creatinine ratio (ACR) (β = 0.11, 95% CI: 0.05–0.18).

The predictive model and its diagnostic performance are illustrated in Fig. 3.

## Discussion

The present study investigated the clinical utility of urinary neutrophil gelatinase–associated lipocalin (NGAL) as a potential early biomarker of diabetic nephropathy in patients with type 2 diabetes mellitus attending Kasr Al-Ainy Hospital, Cairo University. The findings demonstrated a progressive increase in urinary NGAL concentrations with advancing stages of diabetic nephropathy. In addition, NGAL levels showed significant associations with established indicators of renal function, including serum creatinine, albumin-to-creatinine ratio (ACR), and estimated glomerular filtration rate (eGFR). These observations support the concept that urinary NGAL reflects early renal injury and may provide additional diagnostic information beyond traditional markers of diabetic kidney disease.

One of the most notable findings of the present study is the progressive elevation of urinary NGAL across different stages of diabetic nephropathy. Patients with more advanced renal impairment exhibited markedly higher NGAL levels compared with individuals with early disease and healthy controls. This pattern is consistent with previous studies demonstrating that NGAL expression increases in response to renal tubular injury and correlates with the severity of kidney dysfunction [6], [7]. Several investigations have reported similar increases in urinary NGAL among patients with diabetic kidney disease, supporting its potential role as a marker of renal injury in diabetes [8], [9].

An important observation in the current study is that urinary NGAL levels were elevated even in early stages of nephropathy, when conventional markers such as ACR and eGFR may still remain within relatively preserved ranges. This finding supports the growing recognition that diabetic nephropathy is not solely a glomerular disease but also involves early tubular and interstitial injury [4], [5]. Chronic hyperglycemia induces multiple pathogenic mechanisms within renal tubular epithelial cells, including oxidative stress, inflammatory activation, and metabolic dysregulation. These processes may lead to early tubular dysfunction and increased NGAL expression before overt albuminuria becomes detectable.

The significant correlations observed between urinary NGAL and conventional renal biomarkers further strengthen the potential clinical value of this marker. In this study, NGAL showed a positive relationship with both serum creatinine and urinary ACR, while demonstrating an inverse correlation with eGFR. Similar relationships have been described in previous studies evaluating tubular biomarkers in diabetic kidney disease [9], [10]. These findings suggest that NGAL may reflect both early tubular stress and progressive renal dysfunction, thereby complementing traditional markers that primarily reflect glomerular damage.

Receiver operating characteristic analysis in the present study demonstrated that urinary NGAL exhibited excellent diagnostic performance for identifying diabetic nephropathy. Although the observed area under the curve was high in this cohort, interpretation of this result should consider the relatively limited sample size and cross-sectional design of the study. Nevertheless, these findings are consistent with previous research indicating that NGAL may have strong diagnostic potential for detecting early renal involvement in diabetes [8], [10].

The biological characteristics of NGAL provide further explanation for its diagnostic value. NGAL is a small glycoprotein that is rapidly produced by injured epithelial cells and released into the urine in response to cellular stress. In the kidney, tubular epithelial cells markedly upregulate NGAL synthesis following inflammatory or ischemic injury. Because NGAL is released early during the process of tubular damage, its urinary concentration can increase before conventional indicators of renal impairment become abnormal [6], [7]. This mechanism may explain why NGAL elevations were observed in early stages of diabetic nephropathy in the present study.

From a clinical perspective, the identification of reliable biomarkers capable of detecting early renal injury is particularly important for patients with diabetes. Early detection allows clinicians to initiate preventive interventions aimed at slowing the progression of kidney disease, including tighter glycemic control, blood pressure management, and the use of renoprotective pharmacological therapies such as renin–angiotensin system inhibitors and sodium–glucose cotransporter-2 inhibitors. In this context, biomarkers reflecting tubular injury, including NGAL, may provide additional value when used alongside conventional renal markers [11], [12].

The current study also provides data from patients attending Kasr Al-Ainy Hospital, one of the largest tertiary medical centers in Egypt. Investigating emerging biomarkers within different clinical populations is important because genetic, environmental, and lifestyle factors may influence the presentation and progression of diabetic kidney disease. The present findings therefore contribute additional evidence supporting the potential role of NGAL as an early indicator of renal injury in patients with type 2 diabetes.

Despite these promising observations, several limitations should be considered. First, the cross-sectional design limits the ability to determine whether elevated NGAL levels can predict future renal deterioration. Prospective longitudinal studies are required to confirm the prognostic value of this biomarker. Second, the sample size was relatively modest, which may influence estimates of diagnostic performance. Third, serum NGAL was not measured in this study. Combined assessment of serum and urinary NGAL may help differentiate renal-specific tubular injury from systemic inflammatory influences, potentially improving clinical interpretation of NGAL measurements. Finally, although efforts were made to minimize confounding variables through careful inclusion and exclusion criteria, further studies including larger patient cohorts and additional clinical parameters are necessary.

Overall, the findings of this study support the emerging concept that tubular injury plays an important role in the early stages of diabetic nephropathy. Urinary NGAL appears to reflect this early tubular damage and may therefore serve as a useful adjunct biomarker for the detection of diabetic kidney disease. Future longitudinal and multicenter studies are warranted to confirm these findings and to determine how NGAL measurement could be integrated into routine clinical practice for the early identification and monitoring of diabetic nephropathy.

## Conclusion

In summary, this study demonstrated that urinary neutrophil gelatinase–associated lipocalin levels increase progressively with the severity of diabetic nephropathy in patients with type 2 diabetes mellitus. Elevated NGAL concentrations were detectable even in early stages of renal involvement and showed strong associations with conventional markers of kidney function, including serum creatinine, albumin-to-creatinine ratio, and estimated glomerular filtration rate.

These findings support the concept that tubular injury occurs early in the course of diabetic kidney disease and that NGAL may serve as a sensitive indicator of this process. Compared with traditional markers that primarily reflect glomerular dysfunction, urinary NGAL may provide additional diagnostic information by identifying early tubular stress before substantial loss of renal function occurs.

Although further validation in larger prospective studies is required, urinary NGAL shows promise as a non-invasive biomarker that could complement existing screening strategies and potentially facilitate earlier detection and management of diabetic nephropathy.

## CRediT authorship contribution statement

**Elham Yousief:** Funding acquisition, Formal analysis, Data curation, Conceptualization. **Ikram Hassan Adam:** . **Tarek Abdelaziem Ramzy:** . **Ahmed Laymouna:** Data curation.

## Declaration of competing interest

The authors declare that they have no known competing financial interests or personal relationships that could have appeared to influence the work reported in this paper.

## References

[1] Alicic R.Z., Rooney M.T., Tuttle K.R. (2021). Diabetic kidney disease: challenges, progress, and possibilities. Clin J Am Soc Nephrol.

[2] Thomas M.C. (2022). Diabetic kidney disease: Pathophysiology and clinical management. The Lancet Diabetes & Endocrinology.

[3] IDF Diabetes Atlas. (2023). 10th Edition. International Diabetes Federation. 35914061

[4] Papadopoulou-Marketou N. (2022). Early biomarkers of diabetic nephropathy: Focus on tubular injury markers. Diabetes Res Clin Pract.

[5] Navarro-González J.F., Mora-Fernández C. (2021). The role of inflammation in diabetic nephropathy. Nat Rev Nephrol.

[6] Shlipak M.G. (2023). Clinical utility of kidney injury biomarkers in diabetic kidney disease. Kidney International Reports.

[7] Krawczeski C.D. (2021). NGAL as a biomarker of kidney injury: Progress and prospects. Nephrol Dial Transplant.

[8] Coca S.G., Nadkarni G.N., Parikh C.R. (2021). Biomarkers for the early detection of diabetic kidney disease. Curr Opin Nephrol Hypertens.

[9] Kim Y. (2022). Urinary NGAL as an early marker of nephropathy in type 2 diabetes: a meta-analysis. Front Endocrinol.

[10] Mishra J. (2023). Clinical significance of urinary NGAL in early diabetic nephropathy. BMC Endocr Disord.

[11] Chan L. (2022). NGAL as a predictor of renal function decline in diabetic patients. Diabetol Metab Syndr.

[12] Liu Y. (2024). Integration of novel biomarkers into diabetic kidney disease management: current evidence and future directions. Front Med.

